# Comment on Flagel et al.: Sign-tracking as a predictor of addiction vulnerability

**DOI:** 10.1007/s00213-021-05928-2

**Published:** 2021-07-29

**Authors:** Veronika Pohořalá, Thomas Enkel, Dusan Bartsch, Rainer Spanagel, Rick E. Bernardi

**Affiliations:** 1 Institute of Psychopharmacology, Central Institute of Mental Health, Medical Faculty Mannheim, University of Heidelberg, J5 68159 Mannheim, Germany; 2 Department of Molecular Biology, Central Institute of Mental Health, Medical Faculty Mannheim, University of Heidelberg, Mannheim, Germany

Flagel et al., in their Letter to the Editor *Sign-tracking as a predictor of addiction vulnerability*, present a reasoned and valuable comment on our recent study (Pohorala et al. 2021) that outlines the seminal work on sign- and goal-tracking and how these behaviors contribute to drug cue-mediated responding in animals. We concur with most of what the authors have written; however, we do not entirely agree with their seeming conclusion that only a defined set of experimental measurements should be used to uncover cognitive-motivational traits that may underlie phenotypes associated with sign- and goal-tracking. Although not all of the conditions of our study—as Flagel et al. rightfully point out in their letter—were those with which behavioral differences between sign-tracking and goal-tracking have typically been most apparent, it was in fact the objective of our study to specifically evaluate whether a specific phenotype (sign-tracking) demonstrated under a given set of conditions (discrete drug-associated cues) exhibited similar addiction-like behaviors under a different set of experimental conditions (the 3-CRIT protocol). Similarly, it must be emphasized that the value of the sign-tracking phenotype with respect to addiction lies—again as the authors state—in its ability to *predict* addiction vulnerability. And because the 3-CRIT model is, to date, arguably the most translationally relevant multisymptomatic model of addiction available, it stands to reason that phenotypes associated with sign-tracking might, in fact, relate to and predict addictive-like behavior in the 3-CRIT model. In other words, we thought it logical that enhanced cue salience associated with the sign-tracking phenotype might indeed manifest itself in other drug-seeking behaviors, including those measured using the 3-CRIT model. This was hypothesized to be especially relevant considering that 3-CRIT animals have previously demonstrated increased cue and cocaine-induced reinstatement relative to 0-CRIT animals (Cannella et al. 2017, 2013), as well as increased cue-mediated cocaine-seeking following a withdrawal period (unpublished results). These findings are of course consistent with those previously demonstrated in sign-trackers relative to goal-trackers (Saunders and Robinson 2011). Unfortunately, a marriage of these two models, in this experiment under these conditions, examining along the range of Pavlovian approach scores among sign-trackers, intermediates, and goal-trackers, did not result in a correlation with 3-CRIT measures. However, we continue to believe that, given the relevance of the sign-tracking phenotype to vulnerability to addiction-like characteristics in animal models despite limited translational validity, as well as the critical information provided by the 3-CRIT model over the last several years, there may still be a way to bridge these two paradigms. Nonetheless, we are grateful to Flagel et al. for their Letter to the Editor, which provides some welcome further context and nuance to our findings.
